# Mapping Early, Middle and Late Rice Extent Using Sentinel-1A and Landsat-8 Data in the Poyang Lake Plain, China

**DOI:** 10.3390/s18010185

**Published:** 2018-01-11

**Authors:** Haifeng Tian, Mingquan Wu, Li Wang, Zheng Niu

**Affiliations:** 1 The State Key Laboratory of Remote Sensing Science, Jointly Sponsored by Institute of Remote Sensing and Digital Earth of Chinese Academy of Sciences and Beijing Normal University, P.O. Box 9718, Datun Road, Chaoyang, Beijing 100101, China; tianhf@radi.ac.cn (H.T.); wangli@radi.ac.cn (L.W.); 2 College of Resource and Environment, University of Chinese Academy of Sciences, Yuquan Road 19, Shijingshan, Beijing 100049, China

**Keywords:** Sentinel-1A, Landsat-8, remote sensing, rice, classification, synthetic aperture radar

## Abstract

Areas and spatial distribution information of paddy rice are important for managing food security, water use, and climate change. However, there are many difficulties in mapping paddy rice, especially mapping multi-season paddy rice in rainy regions, including differences in phenology, the influence of weather, and farmland fragmentation. To resolve these problems, a novel multi-season paddy rice mapping approach based on Sentinel-1A and Landsat-8 data is proposed. First, Sentinel-1A data were enhanced based on the fact that the backscattering coefficient of paddy rice varies according to its growth stage. Second, cropland information was enhanced based on the fact that the NDVI of cropland in winter is lower than that in the growing season. Then, paddy rice and cropland areas were extracted using a K-Means unsupervised classifier with enhanced images. Third, to further improve the paddy rice classification accuracy, cropland information was utilized to optimize distribution of paddy rice by the fact that paddy rice must be planted in cropland. Classification accuracy was validated based on ground-data from 25 field survey quadrats measuring 600 m × 600 m. The results show that: multi-season paddy rice planting areas effectively was extracted by the method and adjusted early rice area of 1630.84 km^2^, adjusted middle rice area of 556.21 km^2^, and adjusted late rice area of 3138.37 km^2^. The overall accuracy was 98.10%, with a kappa coefficient of 0.94.

## 1. Introduction

Rice is a staple food for more than three billion people worldwide [1,2]. Paddy rice planting areas account for more than 12% of global cropland areas [3] and 40% of the crop yield in China [4]. Paddy rice also plays an important role in some ecological environmental problems, ranging from water use and climate change [5], to disease transmission [6]. Timely and accurate estimation of the area and distribution of paddy rice crops is useful information for governments, planners, and decision makers who formulate policies in terms of food security and ecological sustainability [7,8].

Remote sensing-based techniques are a proven and effective method of estimating the area of paddy rice crops, and may be superior to traditional ground-based surveys [3,7,9]. Many efforts have been made to map paddy rice planting areas by using various classification algorithms and data sources, including optical- and microwave-based remotely sensed data. In terms of classification approaches, they can generally be divided into unsupervised classification [10,11] and supervised classification methods [12,13]. Knowledge- [14] and phenology-based approaches [15] are typical methods used in supervised classification. In terms of optical remote sensing, MODIS and Landsat datasets have been the major data sources used to extract paddy rice extent [8,16,17,18,19]. In addition, several indexes derived from optical remote sensing images have been utilized in previous studies, such as the Land Surface Water Index (LSWI) [20,21], Enhanced Vegetative Index (EVI) [22,23], and Normalized Difference Vegetation Index (NDVI) [24,25]. Microwave-based remote sensing has been increasingly applied to paddy rice mapping as it has advantages that working in all weather conditions regardless of daylight levels. Major datasets used in existing studies include RADARSAT [26], PALSAR [27], and ENVISAT [28].

However, there are several limitations in utilizing remote sensing-based techniques to extract information about paddy rice planting areas: first, uncertainties in optical remote sensing data due to the frequent influence of clouds and cloud shadows during the paddy rice growing season [27]. On an annual basis, more than 65% of days are cloudy in southeast China [6,29,30]. Accordingly, it is difficulty or sometimes impossible for optical sensors to acquire enough cloud-free images during the paddy rice growth stages. Even with its daily revisit frequency, the MODIS satellite system still has difficulty in providing sufficient numbers of good-quality observations for annual paddy rice mapping in moist tropical areas. Additionally, the spatial resolution of MODIS, at less than 250 m, is insufficient for determining the detail paddy rice extent [6,18]. Second, synthetic aperture radar (SAR)-based techniques have been recognized as an attractive alternative for mapping rice crop extent, as radar signals are less affected by cloud coverage and illumination conditions [31,32,33]. However, traditional SAR data availability and cost have limited its agricultural applications. It also has some limitations compared to optical remote sensing, such as a relatively low temporal resolution that cannot detect the multiple growth cycles of rice (e.g., early rice, middle rice and late rice). Third, when using image statistics-based supervised classification methods to extract information on paddy rice, it is difficult and complex to select reliable, representative and all-sided training samples due to spectral variability in different periods and regions, especially at large scales. The major factor is the difference in the phenological characteristics of paddy rice [34]. The natural phenological characteristics of paddy rice are different at large scales. Paddy rice sowing times are variable, leading to different paddy rice phenology at small regional scales. Multiple images in a dataset may have different acquisition dates and spectral variability, although the sowing times were same. Fourth, remotely sensed data redundancy is a serious problem in the time series data that are increasingly used for paddy rice mapping, especially at large scales. This is because the information on some growth stages is not helpful in improving classification accuracy. 

Fortunately, the Sentinel-1A satellite provides a tremendous new opportunity for resolving the abovementioned problems in paddy rice mapping. Sentinel-1A are the next generation of C-band (center frequency: 5.405 GHz) radar sensor with a 12-day revisit time. It was launched by the European Space Agency (ESA) on 3 April 2014 [35,36,37]. The standard L1 product of Sentinel-1A has an interferometric wide-swath mode (IW) with dual polarization (VV/VH), and a spatial resolution of 5 m × 20 m in the range and azimuth directions, respectively. It has an equivalent number of looks of five, and an image resolution of 10 m [38]. This relatively high spatio-temporal resolution provides an outstanding data source for paddy rice mapping. Moreover, the data source is open access.

At present, there are some studies about the extraction of paddy rice information from Sentinel-1A data (S1A). These have demonstrated that S1A’s VH polarization mode is better for paddy rice mapping than its VV polarization mode [31,34,39]. However, the abovementioned problems were not completely solved. For example, a thresholds method was used to extracted paddy rice information for Shanghai, China [39] and these thresholds cannot necessarily be applied to other regions, as backscatter coefficients and spectral characteristics may vary between different regions and periods. 

To resolve these problems, the present study conducted the following research: (1) A new, enhanced, paddy rice information method is proposed that uses S1A to reduce the influence of data redundancy and weather conditions that allows for the possibility of using unsupervised classification methods to precisely extract paddy rice information. (2) Early, middle and late rice crop information was extracted using an unsupervised classification method based on the enhanced paddy rice data. This can resolve the difficulty in selecting reliable, representative and all-sided training samples. (3) Cropland information, such as which annual crop (e.g., paddy rice, maize, tomato, etc.) is planted, was extracted from Landsat-8 data. The cropland information was helpful for improving the accuracy of paddy rice extraction.

## 2. Materials and Methods

### 2.1. Study Area

**Figure 1:**
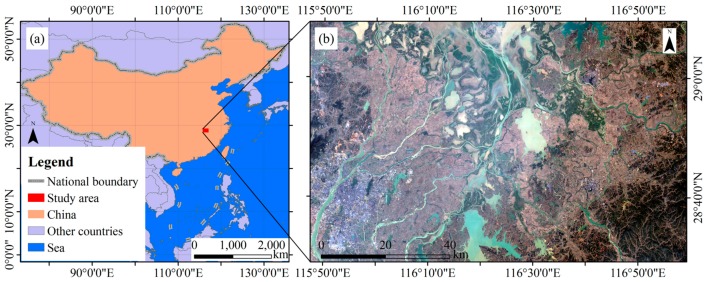
Location of study area: (**a**) The red rectangle indicates the study area (74 km × 120 km) in southeast China; and (**b**) a Landsat-8 true-color image (RGB: 432) of the study area taken on 16 December 2016.

Poyang Lake Plain is locating around Poyang Lake, which is the largest freshwater lake in China. The plains were formed by the alluviation of the Yangtze River and the tributaries of Poyang Lake (e.g., the Xiushui, Ganjiang, Fuhe, Xinjiang and Raohe Rivers). Poyang Lake Plain is the one of the most important areas of paddy rice cultivation in China. Our study site was located in the Poyang Lake Plain and had an area of 74 km × 120 km (Figure 1). In this area, the geographical environment is very complex. The Poyang Lake region has a subtropical monsoon climate that is characterized by a rainy season (April to September) and a dry season (October to March). The mean annual temperature is 25 °C, and the mean annual precipitation is approximately 1600 mm [40].

### 2.2. Sentinel-1A Data and Preprocessing

**Figure 2:**
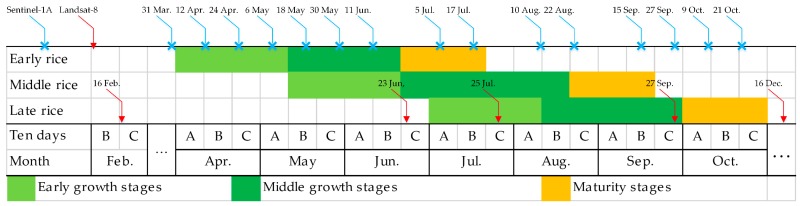
S1A and Landsat image acquisition dates and rice crop calendar in Poyang Lake Plain from early April to late October, showing the early, middle and late stages. The blue sign represents S1A image acquisition dates. The red sign represents Landsat-8 image acquisition dates. The letter “A” represents the first one-third of a month, letter “B” represents the middle third, and letter “C” represents the last third. The early growth stage mainly comprises the sowing and transplanting periods.

Sentinel-1A was launched by the ESA from Europe’s Spaceport in French Guiana on 3 April 2014 [35,36,37]. The S1A IW Level 1 ground range detected and high resolution product, with VH polarization, was downloaded from https://scihub.copernicus.eu/. VH polarization was used because previous studies have demonstrated that VH-polarized backscatter is more sensitive to paddy rice growth than VV-polarized backscatter [31,39]. A total of 15 sets of S1A images (with geo-reference information of UTM zone 50 N, WGS84) were taken from 31 March to 21 October 2016 at 12-day intervals. However, three sets were missing (23 June, 29 July, and 3 September 2016). The spatial dates of S1A image were shown in Figure 2.

Pre-processing of S1A image was performed using the Sentinel Application Platform (SNAP) software (version 6.0) provided by the European Space Agency (ESA) [41]. The workflow included five main steps: (1) S1A images were corrected using orbit files; (2) S1A images were radiometrically calibrated to output *σ°* bands; (3) sigma zero (*σ°*) bands were orthorectified using the Range Doppler Terrain Correction algorithm with Shuttle Radar Topography Mission Digital Elevation Model; (4) the backscattering coefficient (in dB) was acquired from the orthorectified *σ°* band according to the equation 10 × log_10_(*σ°*) [42]; and (5) a median filter with a window size of 5 × 5 pixels was utilized to remove speckle noises [43,44,45].

The S1A’s spatial resolution is 5 m × 20 m in the range and azimuth directions, respectively, although its image pixel spacing is 10 m × 10 m in ground geometry [38]. In additional, speckle noise and the median filtering process lowers the spatial resolution. Accordingly, the spatial resolution of the S1A images was resized to 30 m × 30 m using the nearest-neighbor resampling method. This reduced the size of the dataset and matches the 30 m resolution of Landsat-8 data.

### 2.3. Landsat-8 Data and Preprocessing

The Landsat-8 satellite is equipped with two sensor payloads (Operational Land Imager, OLI, and Thermal Infrared Sensor, TIRS) and was launched on 11 February 2013. The Landsat standard Level 1 products consist of eight multispectral bands with a spatial resolution of 30 m, one panchromatic band with a resolution of 15 m, and two thermal bands with a resolution of 30 m [39,46,47]. There were five sets of OLI images (with geo-reference information of UTM zone 50 N, WGS84) used in this study. These were acquired on 16 February, 23 June, 25 July, 27 September, and 16 December 2016, respectively, and were downloaded from http://earthexplorer.usgs.gov/. The spatial dates of Landsat-8 image were shown in Figure 2.

The re-processing work was as follow: First, the images were calibrated to at-sensor radiance images, and then corrected to surface reflectance images using the Fast Line-of-sight Atmospheric Analysis of Hypercube (FLAASH) tool based on the ENVI software (version 4.8), which is the flagship product of Exelis Visual Information Solutions Company (Boulder, CO, USA). Second, geometric pixel-to-pixel precision correction was implemented based on S1A data using the georeferencing model of ArcGIS software (version 9.2) (Redlands, CA, USA). Finally, all images were cropped to cover the study area.

### 2.4. Auxiliary Data and Accuracy Assessment

Auxiliary data included phenological information and field survey data. Phenological information on paddy rice in the study area was gained from http://www.zzys.moa.gov.cn/, and shows the growth cycle of different species of paddy rice (Figure 2). Paddy rice was divided into early rice, middle rice and late rice according to its planting time. The growth cycle of paddy rice was divided into an early growth stage, a middle growth stage and a maturity stage [31]. The early growth stage is the key period for remotely detecting the presence of paddy rice [6,47]. This is because the cropland is inundated during the early growth stage (the sowing or transplanting period) and SAR is sensitive to water. In the middle growth stage, the paddy rice’s backscattering coefficient will be come to peaks. Finally, in the maturity stage, the paddy rice backscattering coefficient begins to decrease.

**Figure 3:**
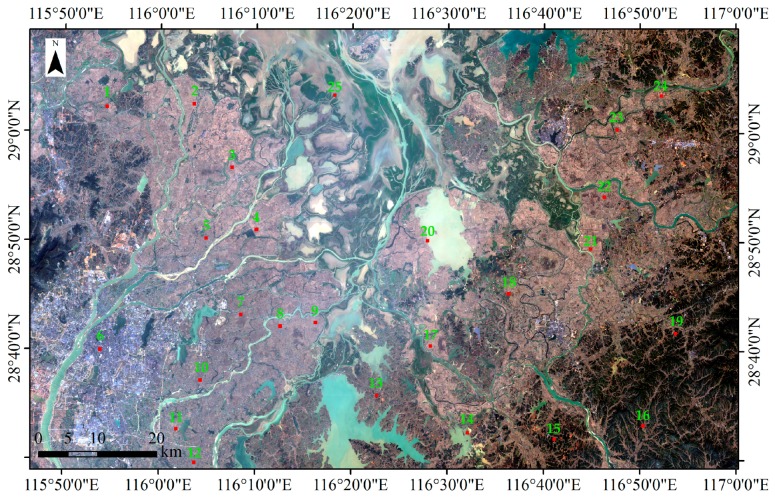
Distribution of field survey quadrats in the study area (red rectangles). The digital with green color was the quadrat number. There were 25 quadrats, each measuring 600 m square. The base map is a Landsat-8 true-color image (RGB: 432) of the study area taken on 16 December 2016.

The field survey dataset comprised 25 quadrats of 600 m × 600 m. Their distribution is illustrated in Figure 3. First, in every quadrat, the boundaries of every surface feature were plotted based on Google Earth images with a spatial resolution of 1 m. Thus, every quadrat was divided into polygons. Second, the land cover and land use attributes of each polygon was defined based on field survey results. Three times field surveys were conducted in the study, during 17–21 May, 10–15 July and 5–9 September 2016. Third, all quadrat polygons were converted to raster data with a spatial resolution of 30 m. These raster datasets were defined as reference classification and were utilized to assess the classification accuracy of paddy rice information extracted in this paper.

There must be some classification errors for each category, thus, the area obtained directly from a classification map may differ greatly from the true area [27,48]. Thus, one of purposes of accuracy assessment was to adjust the classification results. Olofsson et al. [48] had given the spatial accuracy assessment method, as the following. First, the error matrix of sample counts was constructed using the confusion matrix model of ENVI software, as shown in Table 1a. The map categories (*i* = 1, 2, …, *q*) are represented by rows and the reference categories (*j* = 1, 2, …, *q*) by columns in Table 1.

Then the error matrix of estimated area proportion is computed and plotted in Table 1b as the following equation:

(1) $$p_{ij}=\left(A_{m,i}/A_{tot}\right)\times \left(n_{ij}/n_{i\cdot }\right)$$

where *A_m,i_* represents the mapped area of category *i*, and the subscript *m* denotes “mapped”; *A_tot_* represents the total area of the map; *n_ij_* represents the value in Table 1a; and *n_i•_* represents the total of the rows *i* in Table 1a.

An unbiased estimator of the total area of category *j* is computed as the following equation:

(2) $$A_{a,j}=A_{tot}\times p_{\cdot j}$$

where *A_a,j_* represents the adjusted area of category *j*, and the subscript *a* denotes “adjusted”; and *p_•j_* represents the total of the columns *j* in Table 1b.

The estimated standard error of the estimated area proportion is computed as the following equation:

(3) $$S\left(p_{\cdot j}\right)=\sqrt{\sum_{i=1}^{q}{\left(\frac{A_{m,i}}{A_{tot}}\right)}^{2}\times \frac{\left(n_{ij}/n_{i\cdot }\right)\times \left(1-n_{ij}/n_{i\cdot }\right)}{n_{i\cdot }-1}}$$

An approximate 95% confidence interval for the adjusted area of category *j* is,

(4) $$A_{a,j}\pm 1.96\times A_{tot}\times S\left(p_{\cdot j}\right)$$

Last, user’s accuracy (*U*) and producer’s accuracy (*PR*) for any category and overall accuracy (*O*) can be estimated directly from Table 1b according to the following equation:

(5) $$U_{i}=p_{ii}/p_{i\cdot }$$

(6) $$PR_{j}=p_{jj}/p_{\cdot j}$$

(7) $$O=\sum_{j=1}^{q}p_{jj}$$

Kappa coefficient would be given from the classification accuracy report provided by the ENVI software when using the confusion matrix model. For more information about the accuracy assessment method, please refer to Olofsson et al. [48].

### 2.5. Enhanced Paddy Rice Information

The complex natural environment of the study area has an extensive number of rivers, lakes, farms, forests and built-up areas (Figure 1b). Enhancement of paddy rice information is important and is needed to gain better results of paddy rice extraction.

**Figure 4:**
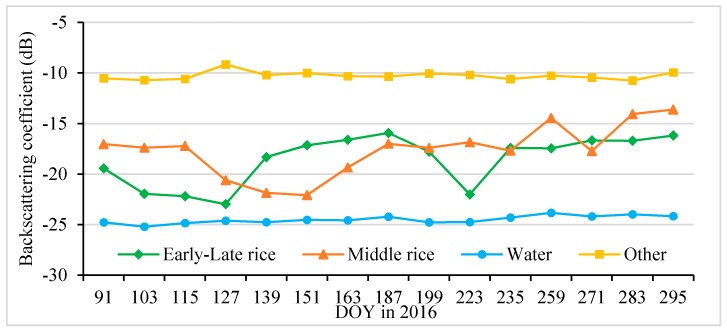
Backscattering coefficients of different remotely-sensed objects. The major objects of interest were early-late rice, middle rice, water and “other” (representing forest and built-up land, which were combined as they had similar coefficients).

The time series of the backscattering coefficients of different objects (including early-late rice, middle rice, water and other) are plotted in Figure 4 according to 427 pure pixels (independent of these 25 quadrats) of these objects. The other objects presented in Figure 4 include forest and built-up land, whose backscattering coefficients were similar. Accordingly, forest and built-up land were categorized as “Other” land use. Figure 4 shows that their backscattering coefficient was approximately −10 (±1) dB on the time series from 31 March to 21 October 2016. In contrast, water’s backscattering coefficient was lower, at −24 (±1) dB. In Figure 4, the lines of both objects’ backscattering coefficients hardly fluctuate over the study period.

**Figure 5:**
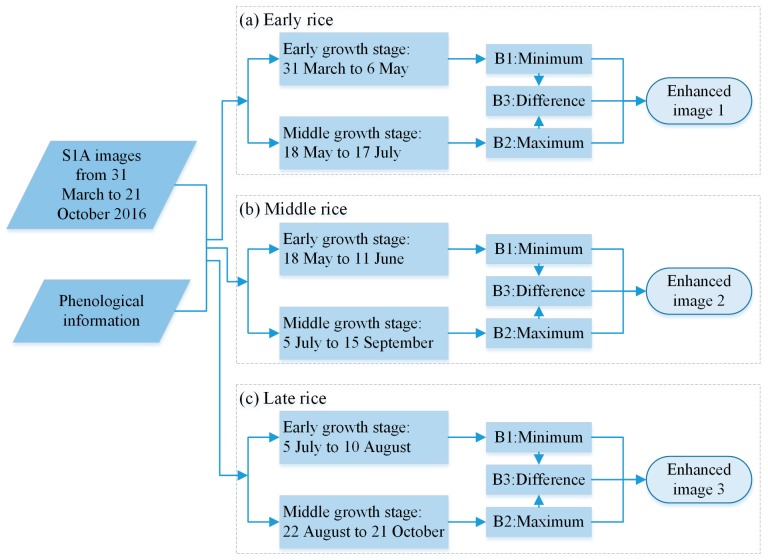
Flow chart of paddy rice image enhancement. “B1: Minimum” was the first band of enhanced image. For each pixel of the first band, its value was the minimum of the image set on paddy rice early growth stage for different types of paddy rice, then all the pixels constituted into the first band. “B2: Maximum” was the second band of enhanced image. For each pixel of the second band, its value was the maximum of the image set on paddy rice middle growth stage for different types of paddy rice. “B3: Difference” was the third band of enhanced image, which was the difference between the first band and the second band.

**Figure 6:**
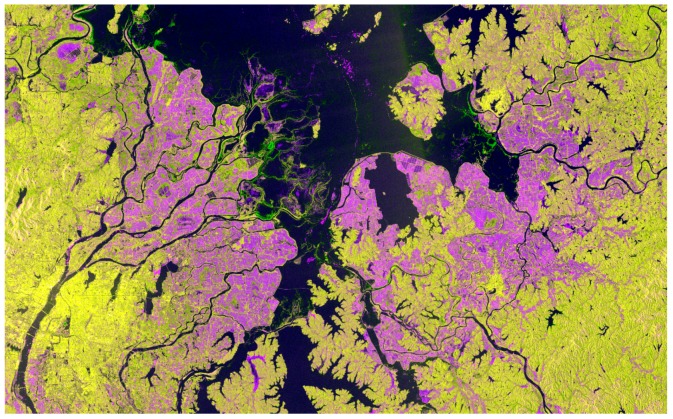
“Enhanced image 1”, where purple is the extent of early rice cultivation, black is water, and yellow is forest and built-up land. Enhanced image 1 is a false color image, where red is the second band (maximum backscattering coefficient band), green is the first band (minimum backscattering coefficient band), and blue is the third band (difference between bands 1 and 2). Enhanced images 2 and 3 are not given here, but are similar to this one.

The paddy rice backscattering coefficients showed distinct fluctuations over the paddy rice growing season. The green line (Figure 4, early-late rice) had two troughs, indicating that the paddy rice backscattering coefficient was low in its early growth stage and peaked in its middle growth stage [31]. Thus, we selected the minimum backscattering coefficient value (early growth stage) as the first band of a new enhanced image, and the maximum value (middle growth stage) as the second band of the new enhanced image. The difference between the first and second bands is regarded as the third band of the new image. The new enhanced image shows enhanced paddy rice information (Figure 5 and Figure 6).

The paddy rice early growth stage was defined as 31 March to 6 May, and the middle growth stage was defined as 18 May to 17 July based on field survey data, phenological information and S1A date. There were four image sets taken by S1A between 31 March and 6 May (Figure 4); therefore, there were four values for each pixel location. The minimum value of these four was extracted to produce the minimum backscattering coefficient band. The maximum backscattering coefficient band was produced in the same way. The “difference band” was calculated as the difference between the maximum and minimum bands. The three bands were stacked into a new image—of enhanced early rice—and named “enhanced image 1” (Figure 5 and Figure 6). Figure 6 shows that the image highlights early rice compared to the other features in the image.

Due to space restrictions, we will not describe the enhancement of middle and late rice in the main text. However, Figure 5 gives the time ranges for the middle and late rice growth stages, which were used to enhance paddy rice information.

### 2.6. Cropland Extraction

Landsat-8 data were utilized to identify cropland areas. Most Landsat-8 data were seriously contaminated due to cloudy and rainy weather; however, there were five good sets of OLI images, which were acquired on 16 February, 23 June, 25 July, 27 September, and 16 December 2016 with percentage cloud cover less than 1%.

The 23 June, 25 July, and 27 September images cover periods when the croplands were covered by green crops. However, there was no green crop in the 16 February and 16 December Landsat-8 images. At these times, the crops would have been harvested, and the cropping areas were bare. The NDVI index is sensitive to vegetation on Landsat-8 images, the vegetation NDVI value is usually greater than the non-vegetation value. This characteristic was used to extract cropland information. The NDVI can be calculated with the following formula:

(8) $$\mathrm{NDVI}=\left(\rho_{nir}-\rho_{red}\right)/\left(\rho_{nir}+\rho_{red}\right)$$

where $\rho_{nir}$ represents the reflectance of the near-infrared band (B5 of OLI) and $\rho_{red}$ represents the reflectance of the red band (B4 of OLI).

The five NDVI bands were computed according to Equation (8) from the five OLI image sets. The maximum NDVI value was selected from the three NDVI bands on 23 June, 25 July, and 27 September and constructed into the maximum NDVI band. Likewise, the minimum NDVI value was selected from the 16 February and 16 December NDVI bands. Then, the difference between the maximum and the minimum NDVI band was regarded as a third band. The three bands were stacked into a new image renamed “cropland-image”. This image highlights cropland and forest information and suppresses other features.

Cropland and forest were extracted using a cropland image-based and unsupervised classifier (K-Means classification) in ENVI software [49,50,51]. The parameter of “number of classes” was set at 10, and the parameter of “maximum iterations” was set at 20, and other parameter settings were default value. Then, the combine classes model of ENVI software was used to combine the same types class according to survey data.

### 2.7. Comprehensive Mapping of Paddy Rice Extent

Early, middle and late rice information was extracted using the three enhanced images (Section 2.5) and K-Means classification in ENVI software. The classification results were named “early rice layer”, “middle rice layer”, and “late rice layer”. There was a large area of water in the study area. We used information on the 2016 water extent from Tian et al. [52] to create the “water layer”. Cropland and forest information from Section 2.6 were named the “cropland layer” and “forest layer”. This created a total of six layers. Each layer has two classifications: there is early rice or non-early rice on the early rice layer, and there is water or non-water on the water layer.

**Figure 7:**
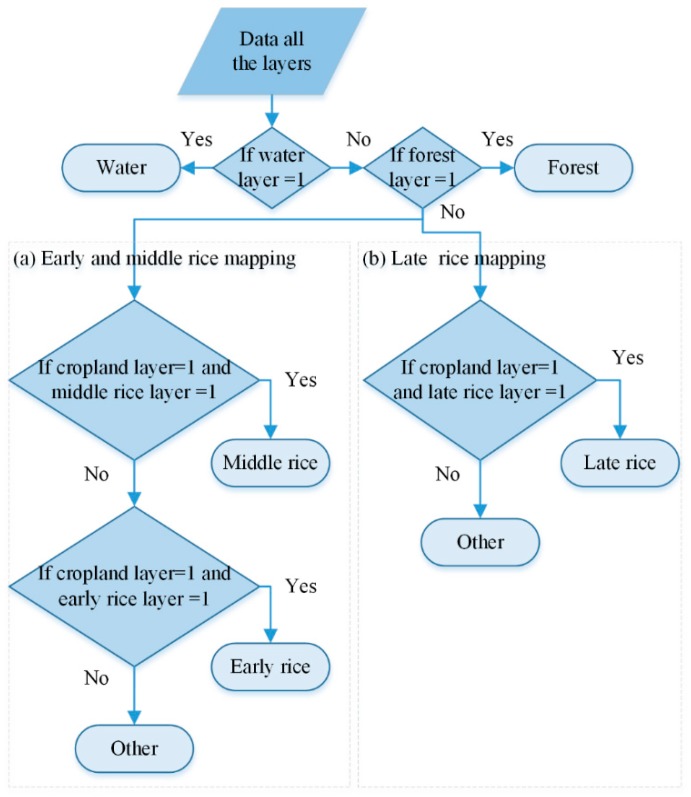
Process used for the comprehensive mapping of paddy rice extent. “Water layer = 1” indicates that the attribute of the pixel was water on the water layer, or the pixel attribute was non-water. The other pseudocode (e.g., “forest layer = 1”, “cropland = 1”) in Figure 7 has the same meaning.

Comprehensive mapping of paddy rice extent was achieved through a decision tree classifier (its pseudo-code is shown in Figure 7). Early and late rice coincided in the same locations, so these objects were plotted on different maps. Comprehensive mapping of early-middle rice extent required four steps: (1) In the first layer of the decision tree, a pixel was regarded as “water” if the pixel was water on water layer and the pixel’s attribute did not change in next layer of the decision tree. (2) In the second layer of decision making, a pixel was regarded as “forest” if the pixel was forest on forest layer and the pixel’s attribute did not change in next decision layer. (3) In the third layer, a pixel was regarded as “middle rice” if it was cropland on cropland layer, and was middle rice on middle rice layer and the pixel’s attributes did not change in next decision layer. (4) In the fourth layer, a pixel was regarded as “early rice” if the pixel was cropland on cropland layer, and was early rice on early rice layer and the pixel’s attributes did not change in next decision layer. The unclassified pixels were divided into “other” land use. Comprehensive mapping of late rice extent was achieved using the same approach.

## 3. Results

### 3.1. Accuracy Assessment

**Figure 8:**
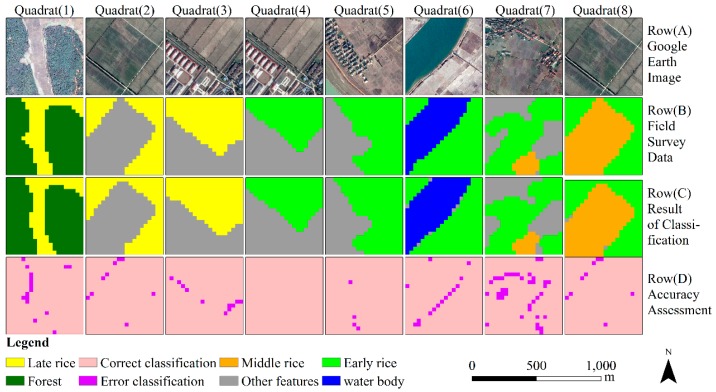
Accuracy assessment of extracted paddy rice information. Quadrats (1) to (8) (each 600 m × 600 m) were used to assess the classification accuracy of extracted paddy rice information. Row (A): Google Earth images from 2016 with 1 m spatial resolution. Row (B): Field survey data with 30 m spatial resolution that were used as ground truth data to assess result of classification. Row (C): Paddy rice classification results (including early, middle and late rice). Row (D): Accuracy assessment. Pink indicates correct classification, and purple indicates errors. Quadrats (2) and (8), (3) and (4) are the same quadrats, however, the sampling times are different.

Classification accuracy was assessed using a confusion matrix approach based on data from 25 field survey quadrats that were 600 m squares. The overall accuracy was 98.10% (±0.28%), with a kappa coefficient of 0.94. The special accuracy parameters for the three types of paddy rice were shown in Table 2. All the user’s accuracy of three types of paddy rice more than 96%, and all the producer’s accuracy of three types of paddy rice more than 95%. To more intuitively or visually show the distribution of classification error, eight results of accuracy assessment were selected randomly from all the accuracy assessment results and were plotted in Figure 8. The paddy rice classification errors are mainly distributing in the boundaries of paddy rice plots and the reason is that there are some mixed pixels.

The early, middle, and late rice unbiased estimator of the proportion of total area was 0.1812, 0.0681 and 0.3487, respectively. The early, middle, and late rice estimated standard error of the estimated area proportion was 0.0033, 0.0014 and 0.0044, respectively.

### 3.2. Paddy Rice Extent

**Figure 9:**
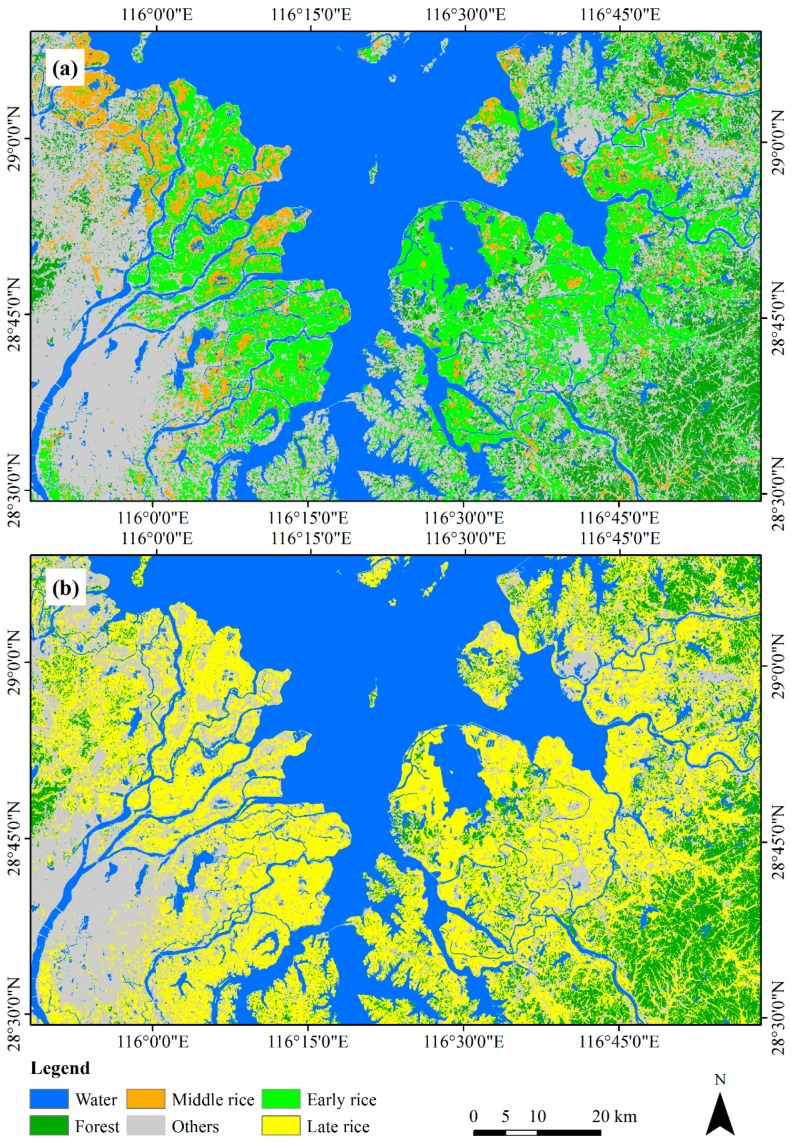
Distribution of extracted paddy rice: (**a**) early and middle rice; and (**b**) late rice.

Early, middle, and late rice extents could be extracted accurately (Figure 9). To accurately compute the area of difference types of paddy rice, the geographic projection of classification map was converted to an Asia North Albers Equivalent Conical Projection with the first standard parallel 25° N, the second standard parallel 47° N, and the central meridian 105° E from UTM zone 50 N [52]. The total area of the study area is 9000 km^2^. The early, middle and late rice area (obtained directly from the classification map) was 1588.39 km^2^, 592.15 km^2^ and 3152.73 km^2^, respectively. These classification areas were adjusted to 1630.84 km^2^, 556.21 km^2^ and 3138.37 km^2^, with a 95% confidence interval of ±58.21 km^2^, ±24.70 km^2^ and ±77.62 km^2^, respectively (Table 3).

Middle rice covered the smallest area of the three types of paddy rice, with 11.10% of the total of paddy rice area, and was mostly distributed in the northwest of the study area. In the early rice season, 25.43% of the areas was middle rice, while 74.57% was early rice. Late rice covered the largest area among the three types of paddy rice, with 59.11% of total of paddy rice area. Compared to the distribution of early rice, there is plenty of late rice in the northwest and southeast hilly regions (Figure 9), and perhaps because of the limitation of temperature. The area of double cropping rice (planting early rice and late rice) was 1583.82 km^2^ (almost equal to the area of early rice), thus one of the purposes of planting early was to plant late rice in this region.

## 4. Discussion

The main contribution of this work is the proposal of a novel approach for identifying early, middle and late rice planting areas using Sentinel-1A and Landsat-8 remotely-sensed data.

The first step of the proposed approach is to enhance the satellite data so that paddy rice areas can be accurately identified using unsupervised classification method. There was high correlation between paddy rice backscattering coefficients and its growth stage and the correlation was the key characteristic to enhance paddy rice information. In the early growth stage, farmland was inundated by water, and the rice seeding was small, so the contribution of water to backscattering energy was greater than that of paddy rice. Water surfaces are smooth and homogenous, causing reflected radar pulses to be weak. Thus, the backscattering coefficient is low in the early stage of paddy rice growth. With growth, the rice plants increasingly obscure the water, and the contribution of paddy rice to backscattering energy was greater gradually than that of water. As the paddy rice canopy is uneven, the reflected radar pulses are more powerful than those reflected from water. Additionally, the volume scattering effect of paddy rice further enhances radar reflection. Thus, the backscattering coefficient becomes high in the middle growth stage, which is why the backscattering coefficient of paddy rice changes distinctly with growth (Figure 4).

Other function of the enhanced paddy rice information major included:

(1) Reducing image data redundancy and compressing image data. For instance, the 15 image bands (668 MB) were reduced to three image sets (342 MB) after enhancement.
(2) Resolving the phenological differences (on the S1A time series) that were caused by differences in planting time and satellite transit time. For example, in one of the images there were two croplands, with planting times of 12 April and 6 May. Therefore, each had different backscattering coefficient dynamics. Such phenological differences present challenges for traditional classification methods.

The order of judgment in Figure 7 was very important. Early and middle rice cannot appear together in the same pixel, because they are mutually exclusive. However, the extraction processes for these both paddy rice is independent of each other, which means that early rice and middle rice may appear together in the same pixel in some places. The backscattering coefficient of middle rice was similar to that of early rice on 6 May 2016 (Figure 4). Thus, some middle rice may be mistakenly divided into early rice in the early rice extraction process. However, early rice could not be divided into middle rice in the middle rice extraction process, because the backscattering coefficient of early rice is much higher than that of middle rice in the early growth stage of middle rice (Figure 4). Thus, we preferentially identified middle rice to resolve this problem (some middle rice may be mistaken for early rice) in Figure 7.

Dong et al. [3] extracted paddy rice with Landsat-8 image in northern Asia, using the phenology- and pixel-based paddy rice mapping algorithm, which identifies the flooding signals in the rice transplanting phase. Nevertheless, the producer’s and user’s accuracy of paddy rice was less than 73% and 93% in their study, respectively. The major reason for the lower accuracy was that the cloud coverage leading Landsat-8 has risks to lose the flooding signals in paddy rice fields. The same issue exists in the study by Kontgis et al. [18]. Thus, it is difficult to accurately extract paddy rice only using Landsat-8 image in Poyang Lake Plain (or tropics and subtropics), because where the rainy weather more frequently compared to that of northern Asia. Nguyen et al. [31] extracted paddy rice using time series Sentinel-1A images and decision tree approach in the Mekong Delta; the producer’s and user’s accuracy of paddy rice were 91.6% and 89.5%, respectively, and the overall accuracy and kappa coefficient were 87.2% and 0.71, respectively. Their accuracy was lower compared to our accuracy. One of the reasons was that these thresholds utilized in decision tree was not enough reasonable, because there were subjective judgments when these thresholds were determined. The other reason was that the Landsat-8 image (optical image, which is sensitive to green vegetation) was not utilized, and only Sentinel-1A image was used, and that would leading to lower user’s accuracy in the study of Nguyen et al. [31].

Additionally, one of purpose of accuracy assessment was to adjust the classification results [18,48]. However, work that adjusting classification results was ignored in some previous studies [31,39]. No matter what classification methods are used, more of less classification errors are unavoidable. Hence, adjusting classification is important to gain a more accurate area.

Although the classification accuracy higher than that of some previous studies [3,18,31], the classification accuracy would be improved further by using more advanced classifier (e.g., random forest classifier [53], deep learning [54]) if we have enough training data.

## 5. Conclusions

This study demonstrates the potential of using Sentinel-1A and Landsat-8 data to accurately map early, middle and late rice cultivation extents. The paddy rice identification method proposed in this paper incorporates the advantages of the Sentinel-1A and Landsat-8 systems. Cropland and forest areas were extracted based on Landsat-8’s sensitivity to green vegetation. Different kinds of paddy rice areas were extracted based on Sentinel-1A’s weather-independent sensors, which are sensitive to the paddy rice growth cycle.

Unsupervised classification was successfully applied in this paper, and could make paddy rice recognition work simple and efficient. Multi-season rice crop remote sensing and monitoring is now possible in rainy areas. This technical approach could be widely applied to monitoring paddy rice—one of the world’s most important food crops.

## Figures and Tables

**Table 1:** Error matrix of sample counts and estimated area proportion.

(a) Error Matrix of Sample Counts (Unit, Pixel)							(b) Error Matrix of Estimated Area Proportion						
	*j*	1	2	…	*q*	Total		*j*	1	2	…	*q*	Total
*i*							*i*						
1		*n*_11_	*n*_12_	*…*	*n*_1*q*_	*n*_1*•*_	1		*p*_11_	*p*_12_	*…*	*p*_1*q*_	*p*_1*•*_
2		*n*_21_	*n*_22_	*…*	*n*_2*q*_	*n*_2*•*_	2		*p*_21_	*p*_22_	*…*	*p*_2*q*_	*p*_2*•*_
⁞		⁞	⁞	⁞	⁞	⁞	⁞		⁞	⁞	⁞	⁞	⁞
*q*		*n_q_*_1_	*n_q_*_2_	*…*	*n_qq_*	*n_q•_*	*q*		*p_q_*_1_	*p_q_*_2_	*…*	*p_qq_*	*p_q•_*
Total		*n_•_*_1_	*n_•_*_2_	*…*	*n_•q_*	*n*	Total		*p_•_*_1_	*p_•_*_2_	*…*	*p_•q_*	1

Map categories are the rows while the reference categories are the columns.

**Table 2:** Accuracy assessment of paddy rice classification.

Accuracy Type	Early Rice	Middle Rice	Late Rice
User’s accuracy (%)	97.93 ± 0.11	98.21 ± 0.05	96.35 ± 0.42
Producer’s accuracy (%)	95.39 ± 0.33	95.92 ± 0.14	96.80 ± 0.44

**Table 3:** Paddy rice area in different types.

Area Type	Early Rice	Middle Rice	Late Rice
Classification area (km^2^)	1588.39	592.15	3152.73
Adjusted area with 95% confidence interval (km^2^)	1630.84 ± 58.21	556.21 ± 24.70	3138.37 ± 77.62
